# Clinical Application of 3D-Printed Patient-Specific Polycaprolactone/Beta Tricalcium Phosphate Scaffold for Complex Zygomatico-Maxillary Defects

**DOI:** 10.3390/polym14040740

**Published:** 2022-02-14

**Authors:** Woo-Shik Jeong, Young-Chul Kim, Jae-Cheong Min, Ho-Jin Park, Eun-Ju Lee, Jin-Hyung Shim, Jong-Woo Choi

**Affiliations:** 1 Department of Plastic and Reconstructive Surgery, Asan Medical Center, University of Ulsan College of Medicine, 88 Olympicro 43 gil, Songpa-gu, Seoul 05505, Korea; woosjeong.ps@gmail.com (W.-S.J.); youngchulkk@naver.com (Y.-C.K.); psdoc87@gmail.com (J.-C.M.); leptonfamily@gmail.com (H.-J.P.); 2 Research Institute, T&R Biofab Co., Ltd., Seongnam-si 13487, Korea; ejlee@tnrbiofab.com (E.-J.L.); happyshim@tnrbiofab.com (J.-H.S.); 3 Department of Mechanical Engineering, Korea Polytechnic University, Sihueng-si 15073, Korea

**Keywords:** polycaprolactone, tricalcium phosphate, PCL/β-TCP, 3D printing, maxillary defect

## Abstract

(1) Background: In the present study, we evaluated the efficacy of a 3D-printed, patient-specific polycaprolactone/beta tricalcium phosphate (PCL/β-TCP) scaffold in the treatment of complex zygomatico-maxillary defects. (2) Methods: We evaluated eight patients who underwent immediate or delayed maxillary reconstruction with patient-specific PCL implants between December 2019 and June 2021. The efficacy of these techniques was assessed using the volume and density analysis of computed tomography data obtained before surgery and six months after surgery. (3) Results: Patients underwent maxillary reconstruction with the 3D-printed PCL/β-TCP scaffold based on various reconstructive techniques, including bone graft, fasciocutaneous free flaps, and fat graft. In the volume analysis, satisfactory volume conformity was achieved between the preoperative simulation and actual implant volume with a mean volume conformity of 79.71%, ranging from 70.89% to 86.31%. The ratio of de novo bone formation to total implant volume (bone volume fraction) was satisfactory with a mean bone fraction volume of 23.34%, ranging from 7.81% to 66.21%. Mean tissue density in the region of interest was 188.84 HU, ranging from 151.48 HU to 291.74 HU. (4) Conclusions: The combined use of the PCL/β-TCP scaffold with virtual surgical simulation and 3D printing techniques may replace traditional non-absorbable implants in the future owing to its accuracy and biocompatible properties.

## 1. Introduction

The management of a maxillary defect is complicated when surgeons must replace the original 3D structure of the bone and carry out functional midfacial restoration in the periorbital and perioral region. Vascularized bone flaps have been the standard option in the field of mandibular reconstruction [1]. They provide a rigid and durable structure that allows adjuvant radiation treatment, a skin paddle for additional soft tissue defects, space for dental implant placement, and reasonable adaptation to remnant bony structures. However, no single flap can provide sufficient volume or support in larger or complex defects, especially when orbital adnexae and dental components are involved.

In complex maxillary treatments, alloplastic material has been combined with autogenous reconstruction. Titanium mesh has been widely applied because it is easy to use and biocompatible, allowing the ingrowth of connective tissue through the implant. Moreover, it can be molded into the complex maxillary structure [2]. However, it can lead to implant exposure or palpability due to the breakdown of the mucocutaneous lining. Deformative change can also occur during scar contracture and adjuvant radiation treatment [3,4]. To address these limitations, biodegradable or bioabsorbable materials have gained popularity; they are rigid and biocompatible, induce bone regeneration, and confer a lower chance of foreign body reaction [5,6,7].

Combined with computer-aided techniques, such as virtual surgical planning, various alloplastic materials have improved the accuracy of maxillofacial reconstruction [8,9,10]. 3D printing technology, combined with preoperative planning and modeling, enables more effective patient-specific treatment. In addition, biodegradable printing materials can now be used in a customized fashion to reconstruct complicated craniomaxillofacial defects with acceptable outcomes. Among these various biodegradable materials, PCL (polycaprolactone) has been used as guided bone regeneration (GBR) membrane owing to its favorable mechanical properties and biocompatibility with a slower degradation rate [11,12]. The beta-tricalcium phosphate (β-TCP), a bioceramic material, has been used in the field of bone tissue engineering owing to its chemical properties resembling bone minerals and excellent osteoconductivity [13,14,15]. The use of PCL blended with beta-tricalcium phosphate (β-TCP) was reported as a promising GBR membrane to promote new bone formation, with an initial stability comparable to cortical bone [11,16,17,18]. Traditionally, promising results were achieved in terms of osteogenic activity when a PCL scaffold was blended with 20% TCP [19,20,21]. To the best of our knowledge, there are few studies using a 3D-printed PCL/β-TCP scaffold in complex zygomatico-maxillary defects. The present work aims to evaluate the new bone formation and 3D conformity using a computed tomographic data and clinical outcomes in zygomatico-maxillary reconstruction with a 3D-printed PCL/β-TCP scaffold.

## 2. Materials and Methods

We evaluated a prospective series of eight patients with complex zygomatico-maxillary defects who underwent reconstruction with 3D-printed PCL implants between December 2019 and June 2021. The inclusion criteria were as follows: (1) unilateral zygomatic maxillary defect with or without orbital floor involvement, (2) maxillary defect resulting from cancer ablation, benign tumor resection, trauma, or degenerative change of the hemiface such as Parry–Romberg syndrome, (3) requirement of immediate or delayed reconstruction due to maxillary defect, and (4) follow-up period of at least six months. The exclusion criteria were as follows: (1) bilateral defect, (2) critical infectious disease or immune deficiency, (3) current or anticipated chemotherapy or immune suppression therapy, and (4) pregnancy or possibility of pregnancy.

Demographic information regarding sex, age, underlying disease, cause of defect, onset of reconstruction (immediate or delayed), type of maxillary defect, and postoperative complications were reviewed. The maxillary defects were categorized based on the amount of vertical and horizontal maxillary defect, as suggested by Brown et al. [22]. Surgical details regarding reconstructive options, incisional approach, application of bone forming material, implant fixation method, and revisional operation were described. Each patient underwent computed tomography (CT) scans with a slice thickness of 0.6 mm at three time periods, including before surgery and six months after.

This study was conducted according to the Declaration of Helsinki and approved by the independent Ethics Committee/Institutional Review Board of the Asan Medical Center (approval number: 2021-1292), with written informed consent obtained from all patients

### 2.1. 3D Simulation and 3D Printing of Patient-Specific Implants

**Figure 1:**
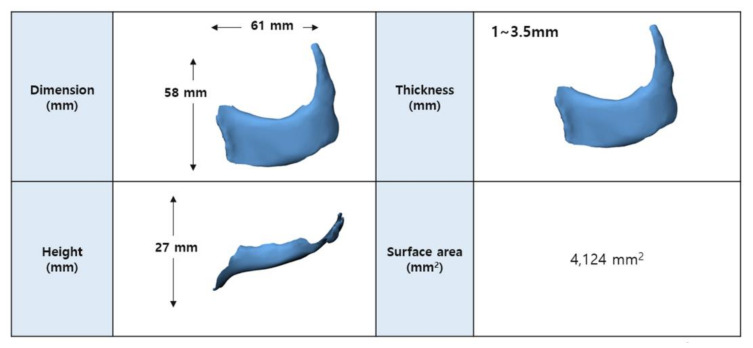
Design of patient-specific PCL/β-TCP scaffold in patient #1. The implant was three-dimensionally designed using 3D modeling software based on the mirror imaging of a contralateral normal zygomatico-maxillary structure.

The patient-specific implants were designed using 3D modeling software (Materialise Mimics; Materialise NV, Leuven, Belgium). The anticipated maxillary defect was marked on a stereolithography model of the skull, and the contralateral normal orbit was flipped to obtain the ideal normal contours of the defect. A patient-specific implant was designed over the region of interest, fabricated and then refined, with smoothing of the contour (Figure 1). All processes were performed under close communication between modeling experts and plastic surgeons.

**Figure 2:**
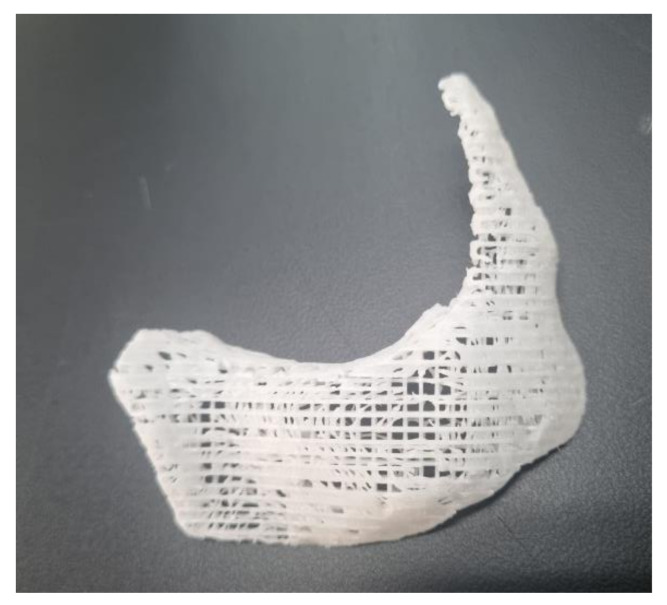
Photograph of 3D printed PCL/β-TCP scaffold with line with 500 μm, and 50% of porosity.

The PCL (Evonik Industries, Essen, Germany) and β-TCP (Foster corporation, Putnam, CT, USA) were mixed in a ratio of 8:2. After the PCL was melted by heating for 15 min at 110 °C, powdered β-TCP was added, which was then blended for 10 min. The PCL/β-TCP mixture was 3D printed using a multi-head deposition system using computer-aided manufacturing software. It had a rectangular pore architecture with a porosity of 50% and a pore size of 500 μm, as determined by 3D modeling software (3-Matic Research 9.0, Materialise, Leuven, Belgium). The scaffolds were freeze-dried at −85 °C for 24 h, then sterilized under a 450 W UV lamp for 4 h. All manufacture process was managed by a facility with Good Manufacturing Practice certification (T&R Biofab Co., Ltd., Seoul, Korea). The image of the 3D printed scaffold is depicted in Figure 2. 

### 2.2. Surgical Procedure

Patients presented with a wide range of maxillary bone and soft tissue defects of various etiologies. The bone defect area was exposed as much as possible so that the implant could be inserted. The bone defect was covered with the 3D-printed implant, with or without osteocutaneous free flaps. The PCL implant was embedded in a betadine solution for 10 min before insertion. If necessary, it was easily molded using a No. 15 blade or scissors, depending on the actual defect. After the PCL implant was inserted into the defect, it was fixed to the adjacent bony structure using mini-plates and 6–8 mm titanium screws. Additional free flaps were indicated if the alloplastic implant necessitated soft tissue envelop to cover the defect. Immediate adverse reactions related to the implant, such as allergic reactions, were checked during surgery.

### 2.3. Volume and Density Analysis Based on CT Data

**Figure 3:**
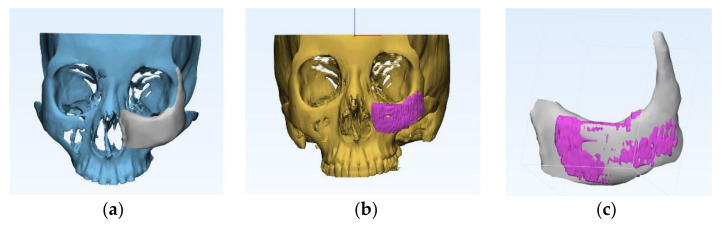
Volumetric analysis between preoperative planned model and actual surgical result. (**a**) Preoperatively designed STL model. (**b**) Actual surgical result volume-rendered as STL model. (**c**) Two images were superimposed based on anatomic landmarks. Overlapping between the simulated implant volume and postsurgical implant volume was calculated using the Boolean operation.

A CT scan was performed before surgery and six months after to evaluate volumetric and density change. The DICOM data were translated into a stereolithography model in 3D modeling software (Mimics; Materialise Software Solutions, Leuven, Belgium) to simulate a postoperative image using a volume rendering technique. The region of interest was defined before surgery along the contour of the simulated implant object, as well as six months after surgery along the outer surface of the inserted implant. Two images were superimposed based on anatomical landmarks, including the anterior nasal spine, nasion, gonion, and menton. Overlapping between the simulated implant volume and postsurgical implant volume was calculated using the Boolean operation. The volume conformity was defined as the percentage of overlapping volume between the simulated and postsurgical images (Figure 3).

**Figure 4:**
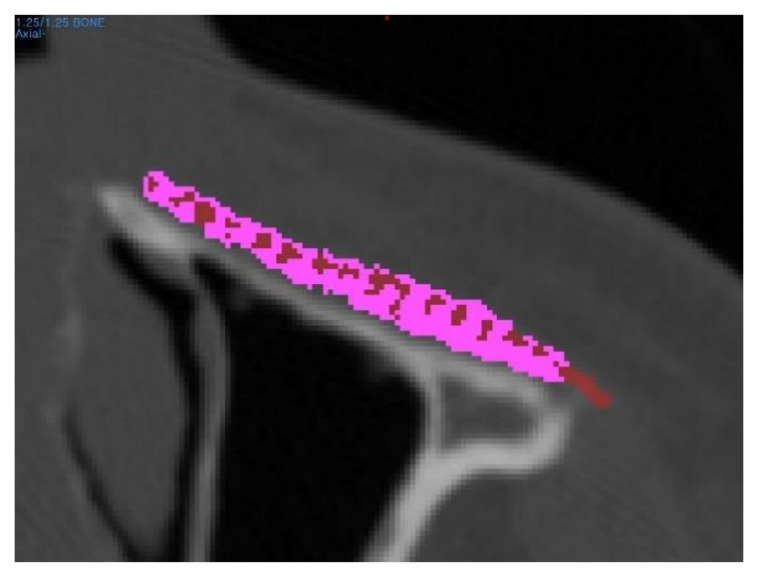
The bone volume fraction was defined as a ratio of the de novo bone volume to the total implant volume within the region of interest. Red area notes the region where the tissue density was measured over 200HU, while the purple area denotes the PCL/β-TCP scaffold.

To identify de novo bone formation, the CT images were subjected to radiodensity analysis using a 3D modeling software (Mimics, Materialise Software Solutions, Leuven, Belgium); the radiodensity was measured in Hounsfield units (HU) in the region of interest. The applied threshold to measure the bone mineral density of newly regenerated bone was 200 HU. The bone volume fraction was defined as the volume ratio of de novo bone to the total implant within the region of interest (Figure 4). In addition, the mean tissue density of the region of interest was investigated at different time periods, including before surgery and six months after surgery.

### 2.4. Tensile Test of the Scaffold

Tensile testing was performed using a single column universal testing machine (Instron, Norwood, MA, USA). The dimension of the scaffold sample was standardized to 10 × 40 × 1 (mm), and porosity was 50%. The number of the sample for the test was 7. The Young’s modulus was calculated by the linear curve of the stress–stain curve.

## 3. Results

Eight patients were included in this study, presenting a wide range of maxillary defects of various etiologies. The causes of the defects were as follows: intraosseous hemangioma in two patients, immediate reconstruction following cancer ablation in three patients, and Romberg disease, traumatic facial deformity, and fibrous dysplasia in one patient each. Five of the eight patients underwent immediate reconstruction following tumor ablation, while three underwent delayed reconstruction. There was a case of wound dehiscence caused by partial flap necrosis, which required wound coverage by a local flap. Detailed information regarding demographics are depicted in Table 1.

Regarding surgical details, in four of the eight patients, the 3D-printed implant was inserted through a perioral and conjunctival incision. The other four patients underwent concurrent free flap or free bone grafts. In patients who had undergone cancer ablation, a head and neck surgeon used lateral rhinotomy and a Weber–Ferguson incision. A bone-forming substance was used in three patients: a demineralized bone matrix (DMB) in two patients and a demineralized calcium phosphate bone substitute in one patient. Revisional operation was required in four patients who underwent a secondary fat graft and one patient who underwent local wound coverage to treat partial flap necrosis (Table 2).

The result of the volume analysis was as follows. The mean preoperatively planned implant volume was 11.32 mm^3^, ranging from 2.16 mm^3^ to 30.37 mm^3^. The mean postoperatively actual implant volume was 10.21 mm^3^, ranging from 1.84 mm^3^ to 28.22 mm^3^. After the superimposition of two images, the mean volume conformity was 79.71%, ranging from 70.89% to 86.31%. Postoperatively, the de novo formation of bone was calculated and the mean was 2.15 mm^3^, ranging from 0.22 mm^3^ to 7.15.mm^3^. The bone volume fraction was obtained as the ratio of de novo bone volume and postoperative implant volume, with a mean of 23.34%, ranging from 7.81% to 66.21%. Mean tissue density in the region of interest was 188.84 HU, ranging from 151.48 HU to 291.74 HU (Table 3).

In the mechanical property test, the Young’s modulus of the standardized scaffold with 50% porosity was 162.7 ± 12. 8 MPa (Table 4).

### 3.1. Case Presentation

Representative cases with clinical pictures are described in this section. 

#### 3.1.1. Case 1 

**Figure 5:**
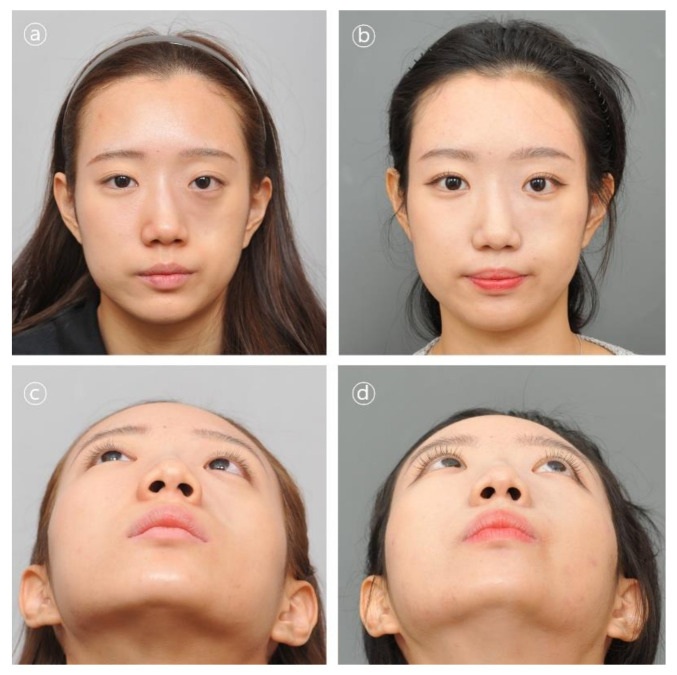
Clinical photographs in patient #1. Contour and symmetry of left cheek region was restored. (**a**,**b**) Pre- and postoperative 6-month frontal view photographs. (**c**,**d**) Pre- and postoperative 6-month basal view photographs.

**Figure 6:**
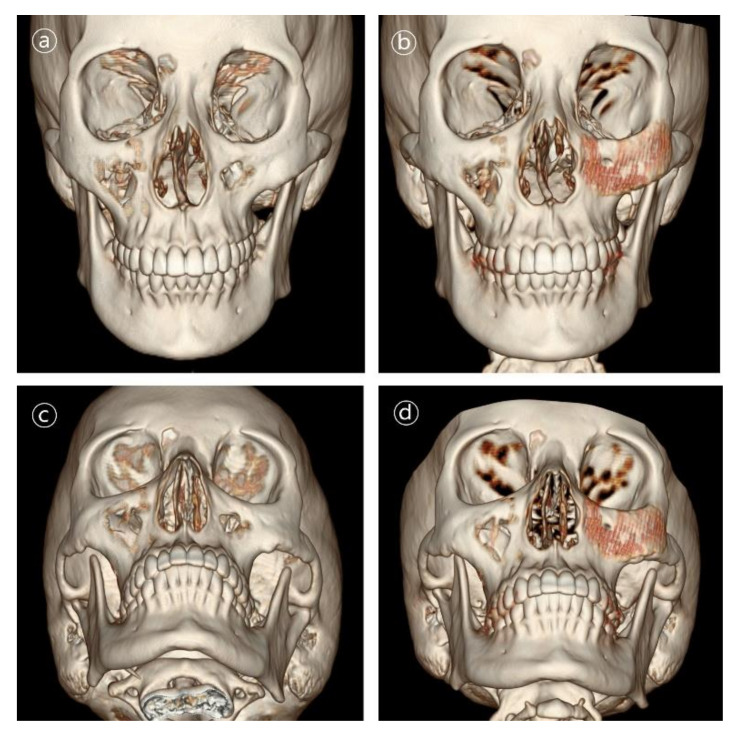
3D CT images in patient #1. Contour and symmetry of left zygomatico-maxillary region was restored with de novo bone formation. (**a**,**b**) Pre- and postoperative 6-month frontal view CT images. (**c**,**d**) Pre- and postoperative 6-month basal view CT images.

Patient #1 was 21-year-old female who underwent delayed reconstruction 24 months after ablation of intraosseous hemangioma. The maxillary bone defect was exposed using the gingivobuccal and transconjunctival approaches. A 3D-printed PCL/β-TCP scaffold was fitted into the defect, and the patient required no further resection of the bony structures. The implant was fixed using a resorbable plate and screws made of HA-PLLA (hydroxyapatite/poly-l-lactide (Figure 5 and Figure 6).

#### 3.1.2. Case 2

**Figure 7:**
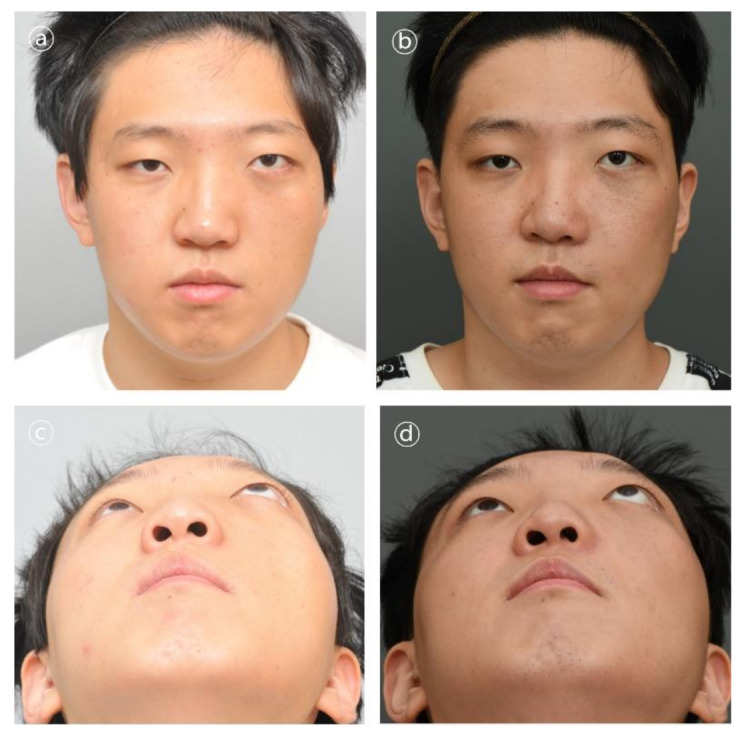
Clinical photographs in patient #5. Contour and symmetry of left cheek region was improved. (**a,b**) Pre- and postoperative 6-month frontal view photographs. (**c,d**) Pre- and postoperative 6-month basal view photographs.

**Figure 8:**
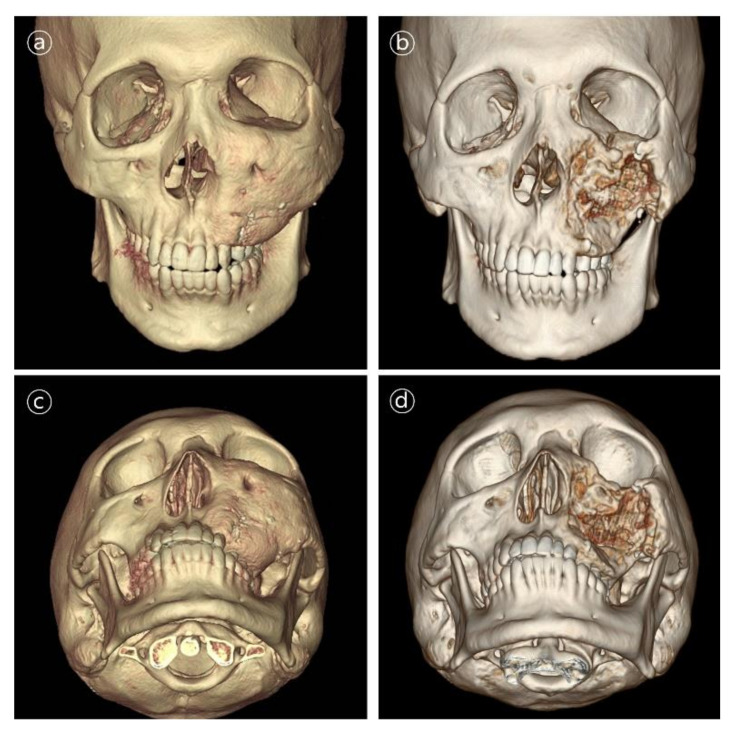
3D CT images in patient #5. Contour and symmetry of left zygomatico-maxillary region was improved. (**a,b**) Pre- and postoperative 6-month frontal view CT images. (**c,d**) Pre- and postoperative 6-month basal view CT images.

Patient #5 was 21-year-old male who underwent immediate reconstruction following the en bloc resection of maxillary fibrous dysplasia, defined as a type V defect. The patient underwent reconstruction with the 3D-printed PCL/β-TCP scaffold through a conventional gingivobuccal and transconjunctival incisions. The 3D-printed implant was fixated with wire steel. There was no complication in the long-term follow-up (Figure 7 and Figure 8).

## 4. Discussion

Polycaprolactone (PCL) is one of the polymers prepared by ring opening polymerization of ε-caprolactone using a variety of catalysts. It safely degrades into carbon dioxide and water over 2–3 years and provides a suitable scaffold for guided bone regeneration [23,24]. The PCL/β-TCP scaffolds used in this study had a 3D shape, moderate rigidity, and relatively high elasticity and were manufactured with a patient-specific design. This property allows surgeons to manipulate and mold the implants using a blade or scissors. In our mechanical property test, Young’s modulus of the scaffold with 50% porosity was 162.7 ± 12. 8 MPa, which is a similar level to that of the human mandibular trabecular bone (6.9 to 199.5 MPa) [25]. It was strong enough to maintain a three-dimensional shape when applied to clinical practice, and also had an adequate elasticity to be carved using tools available in the operating room. However, this might be insufficient to mimic the compressive strength and modulus of cortical bone itself [26,27]. Thus, the characteristics of PCL/β-TCP should be carefully considered depending on the amount of bony defect and surrounding soft tissue. The scaffold might be insufficient to be applied alone in the reconstruction of the whole zygomatico-maxillary complex. However, it was sufficient to bear the tension and compression during biomechanics of the upper jaw as when indicated as an onlay graft onto the bony surface or interpositional graft between the bony gaps. Overall, we did not find any bony instability or occlusal complication during the follow-up period. We suggested that the loading force should be distributed to the underlying bony strut through secure fixation with titanium screws and to overlap with the surrounding bony structure.

The PCL scaffold has been widely used in craniofacial reconstruction of various forms, including mesh, membrane, plate, and 3D implants [28,29,30,31]. Several authors have used PCL mesh in rhinoplasty to replace autogenous cartilage grafts [32]. They have reported that PCL mesh with a 3D structure was a safe and effective material and that it could maintain volume without any foreign body reaction [28]. However, unlike our study, PCL implants in the previous literature have only been applied to 2D reconstruction. Recently, Han et al. used 3D PCL implants in three cases of maxillary reconstruction following cancer ablation. All patients showed favorable outcomes. No signs of infection were observed in any of the three patients, and the existing native tissue was successfully fused with filling of the pores. So far, there has been few reports on the combined use of PCL and β-TCP as a 3D scaffold in clinical cases. We applied a patient-specific PCL/β-TCP scaffold to treat various maxillary defects with a range of etiologies, including facial asymmetry due to Romberg’s disease and ablation of fibrous dysplasia and hemangioma. Notably, we performed a more structured analysis in our cases, measuring volume conformity and bone density. 

Regarding the volume conformity, suboptimal results were obtained in two of the eight cases who underwent immediate reconstruction following maxillary sinus cancer ablation. Although we designed the implants with a 3D shape following the resection plan, the design did not always fit the actual resection margin. This resulted in less conformity between the preoperative simulated and postoperative actual implant volumes. However, experienced head and neck surgeons were fully capable of adjusting the shape of the implants because the material had elastic properties.

Meanwhile, our study reported a case of implant exposure in a patient who had undergone radiation treatment. We reasoned that the wound dehiscence had resulted from delayed wound healing in the irradiated field, especially in the naso-orbital region, rather than from the implant itself. It follows that the implant should be covered with a durable and thick flap, especially when patients have undergone previous radiation, and that meticulous debridement of remaining unhealthy tissue should be carried out to avoid wound complications.

Another complication of the biomaterial that should be considered is the possibility of an allergic reaction. Some rare complications have been reported with the use of biodegradable material due to the wide range of foreign body reactions [33,34,35]. Although there was no allergic reaction reported in our cases, the use of PCL might lead to serious foreign body reactions. Some researchers reported on long-term, late-onset inflammatory complications including granuloma formation, late allergic reaction and chronic inflammation after dermatologic application of PCL-based fillers [36,37]. This reaction seemed to result from an immune overreaction of the host tissue to the product, which is related with underlying inflammatory status of the patient. Thus, the safety of the PCL/β-TCP scaffold in our cases should be proven in the long-term study

In our previous research, we reported on the three-dimensional internal structure of a scaffold using 3D printing [26,38,39,40]. In the case of our 3D printed scaffold, it has an internal structure in which pores with a size of several hundred micrometers are completely interconnected by a layer-by-layer fabrication method. When implanted into the body, these perfectly connected pores are advantageous for the penetration of surrounding cells, and also help the engraftment of regenerated tissue inside the artificial scaffold as blood vessels are connected. 

The effect of the material composition and porosity of a scaffold on its properties, including cell proliferation and differentiation, stiffness, and degradation, has been discussed in the literature [11,41,42,43,44,45,46,47]. The addition of β-TCP in PCL was shown to improve the scaffold’s mechanical performance and increase osteogenic cell proliferation and differentiation [41,42]. By increasing the β-TCP concentration in the scaffolds, significantly higher mineralization was achieved compared to the pure PCL [48]. In addition, the bioceramic composition in the PCL scaffold was shown to increase water absorption and induce hydrophilic properties, which can be useful to prevent nutrient loss during bone regeneration [45]. Other considerations are the porosity, pore size, and permeability of the scaffold, which plays a significant role in biological delivery and tissue regeneration [11,46,47]. Larger pore size and porosity could be beneficial for bone tissue growth but may affect the compressive strength and modulus of the scaffold. Bruyas et al. found that both an increasing amount of β-TCP and decreasing porosity augmented the modulus of the 3D printed scaffolds, while decreasing the elasticity [43]. 

In our experience, when the amount of β-TCP in PCL is increased, viscosity also increases, and as PCL/β-TCP blend viscosity affects scaffold printing speed, 3D printer feed rate reduces, and the polymer is exposed to more thermal energy. When the weight proportion of β-TCP in PCL was more than 20% and the pore size was set to larger than 500 μm, we observed that the printing accuracy and mechanical strengths decreased. Thus, we used the PCL/β-TCP scaffold with a ratio of 80:20 and pore size of 500 μm to achieve balance between β-TCP content and printing rate. 

The degradation profile of the scaffold is another factor that should be considered. The PCL has extremely slow progress of degradation, ranging from 2 to 4 years, while the TCP has an unpredictable biodegradation profile, ranging from 6 to 24 months [44,49,50]. In general, it was reported that the PCL/β-TCP composites had a faster degradation rate than that of pure PCL. Yeo et al. reported the PCL–20% TCP scaffold gradually degraded within 6 months, while maintaining its pore interconnectivity for newly mature bone to form [24]. Initial degradation of β-TCP can produce calcium ions and enhance mineralization, thereby promoting osteogenic differentiation of adipose-derived stem cells. Bruyas et al. found that higher ceramic content of over 40% TCP might lead to structural integrity of the scaffold due to the extremely high rate of degradation [43]. We agreed on their opinion in that such a manipulation of the ceramic ratio to create an ideal bioresorbable plate to match the natural healing course of bone formation. From CT findings obtained during the six-month follow-up of clinical cases, we judged that the 80:20 proportion of PCL:β-TCP and 500 µm pore size of the implant were adequate to enhance earlier bone growth and maintain durability. Other animal studies also corroborated this view, reporting neovascularization, sufficient soft tissue ingrowth, and the absence of extensive inflammation with this pore size and porosity [49].

We concluded that bone regeneration was confirmed based on CT scan results six months after surgery. In particular, it was based on the bone mineral density value from the CT image. We thought that the bone mineral density value reflected not only the purely regenerated bone but also the density of the implanted scaffold as well. However, due to the radiolucent characteristic of the biodegradable polymer, the contribution to the bone mineral density value is insignificant. Nevertheless, histological analysis from the biopsy tissue might be required for confirming the obvious bone regeneration, but it has limitation due to ethical issues. On the other hand, according to a previous study conducted by our research team, an obvious bone regeneration result was confirmed eight weeks after transplantation in an animal experiment using the same PCL/TCP scaffold applied in this study [51]. 

We used various materials, including a mixture of demineralized bone matrix and blood controlled thermal responsive polymer. Demineralized bone matrix has been widely used as a mixture material to enhance bone union and new bone formation [52]. Various artificial materials, including oxidized-irradiated alginate hydrogel and hydroxyapatite were combined with the 3D scaffold. Some authors have reported the combined use of bone morphogenic proteins (rhBMP-2) to treat mandibular defects [50]. However, we should be reluctant to apply this material in patients who have undergone cancer ablation as it is unclear whether rhBMP-2 promotes or inhibits tumor generation [53].

The present study had the following limitations: (1) As we assessed the density in a region of interest containing both the implant and new bone, we did not obtain the actual bone density, which might be lower than the normal bony structure outside of the implant; (2) Although a degradation period from 2 to 4 years for PCL and 6 to 24 months for TCP are known, the speed of degradation will vary depending on the transplant site due to characteristic of hydrolysis. Therefore, a long-term follow-up of more than 5 years is required for future studies; (3) The measured efficacy of PCL mesh in bone formation may have been confounded because we also applied osteoblastic agents. In the present study, we could not assess the efficacy of the combined mixture substances for bone formation, as we performed no comparative analysis. More structured investigation is necessary, with a prospective, comparative, controlled design.

## 5. Conclusions

The PCL/β-TCP scaffold can provide durable support and enhance bone formation in complex zygomatico-maxillary defects. The combined use of virtual surgical simulations, 3D printing techniques, and biodegradable implants may replace traditional non-absorbable implants because the method is more accurate and the materials more biocompatible.

## Figures and Tables

**Table 1:** Demographics of patients.

	Sex	Age	Cause of Defect	Location	Type of Defect	Onset of Reconstruction	Postoperative Complication	Underlying Disease
Patient #1	F	21	Intraosseous hemangioma	Rt.	N.A.	24-month delayed	None	None
Patient #2	M	19	Romberg disease	Rt.	N.A	Delayed	None	None
Patient #3	M	51	Intraosseous hemangioma	Lt.	V	Immediate	None	None
Patient #4	F	50	Traumatic facial deformity	Lt.	N.A	60-month delayed	None	None
Patient #5	M	21	Fibrous dysplasia	Lt.	IIIb	Immediate	None	None
Patient #6	F	43	Radiation necrosis following nasal cavity cancer ablation	Lt.	IIIb	Immediate	Wound dehiscence due to delayed wound healing	Diabetes
Patient #7	F	44	Radiation necrosis following maxillary sinus cancer ablation	Lt.	IIIb	Immediate	None	Hypertension
Patient #8	M	42	Maxillary sinus cancer	Rt.	V	Immediate	None	None

N.A.: Not applicable.

**Table 2:** Surgical details.

	Reconstructive Option	Incisional Approach	Application of Bone Regeneration Material	Implant Fixation	Revisional Operation
Patient #1	Fat graft	Gingivobuccal and transconjunctival	None	HA-PLLA resorbable plate and screws	Secondary fat graft
Patient #2	Fat graft	Gingivobuccal and transconjunctival	Resorbable calcium phosphate bone substitute	Titanium miniplate and screws	Secondary fat graft
Patient #3	Fat graft	Gingivobuccal and transconjunctival	DBM	Titanium miniplate and screws	Secondary fat graft
Patient #4	Fat graft	Gingivobuccal and transconjunctival	None	Titanium miniplate and screws	Secondary fat graft
Patient #5	Iliac bone graft	Gingivobuccal and transconjunctival	DBM	Wire steel	None
Patient #6	RFFF, Iliac bone graft	Weber-Ferguson approach	None	Titanium miniplate and screws	Local wound coverage
Patient #7	ALT FF, RFFF	Lateral rhinotomy and subcillary approach	None	Titanium miniplate and screws	None
Patient #8	None	Lateral rhinotomy and subcillary approach	None	Wire steel	None

HA-PLLA: Hydroxyapatite/poly-l-lactide; DBM: Demineralized bone matrix; ALT FF: Anterolateral thigh free flap; RFFF: Radial forearm free flap.

**Table 3:** Volume and density analysis.

	Preoperatively Planned Implant Volume (mm^3^)	Postoperative Actual Implant Volume (mm^3^)	Conforming Volume after Superimposition (mm^3^)	Volume Conformity (%)	Postoperative Newly Generated Bone Volume (mm^3^)	Bone Volume Fraction (%)	Postoperative Mean Tissue Density (HU)
Patient #1	11.82	10.55	9.62	81.39	1.25	11.87	165.55
Patient #2	8.76	8.42	7.51	85.77	3.15	37.41	184.22
Patient #3	3.72	3.22	2.64	70.89	0.25	7.81	223.00
Patient #4	2.16	1.84	1.66	76.76	1.22	66.21	291.74
Patient #5	30.37	28.22	26.22	86.31	7.15	25.34	184.55
Patient #6	15.88	13.51	11.53	72.59	2.13	15.73	168.44
Patient #7	2.74	2.49	2.16	79.05	0.22	8.80	151.48
Patient #8	15.09	13.42	12.82	84.96	1.82	13.54	182.51

HU: Hounsfield unit.

**Table 4:** Experimental result of mechanical property test.

Scaffold Dimension (mm)	Porosity (%)	Young’s Modulus	Number of Sample
10 × 40 × 1	50	162.7 ± 12. 8 MPa	7

## Data Availability

Not applicable.
